# Metformin as a Fetal Hemoglobin Inducer in Non-transfusion Dependent Thalassemia Patients

**DOI:** 10.1007/s12288-023-01662-1

**Published:** 2023-05-05

**Authors:** Mona ElTagui, Mona El-Ghamrawy, Shareef Karam AlDeeb, Mariam Saad Nassim

**Affiliations:** Department of Paediatrics, Kasr Alainy Faculty of Medicine, Cairo University, 42 Ali Ibrahim Ramez Street, Behind Heliopolis Hospital, El Nohza, Cairo, Egypt

**Keywords:** Metformin, Non transfusion dependent thalassemia, Hydroxyurea, HbF

## Abstract

Non-transfusion dependent thalassemia (NTDT) refers to a group of thalassemic disorders who do not need regular transfusions for survival, however it may be needed in certain conditions. Metformin was reported as a potential fetal hemoglobin (HbF) inducing agent in vitro but its efficacy and safety in vivo was not fully studied. This is a prospective interventional study aimed at studying the effect of metformin on HbF change in NTDT. Methods: Patients with established diagnosis of NTDT were enrolled. They were receiving a stable fixed dose of Hydroxyurea over the last 3 months. Patients were divided into two groups: a group that received Metformin for 6 months (Metformin group) and a control group. Complete blood picture, reticulocytic count, hemoglobin electrophoresis, liver enzymes, bilirubin, kidney functions, LDH and random blood sugar were performed at onset, 3 and 6 months of the study. All adverse events were recorded. Results: Forty two patients aged 12–23 years were enrolled. Metformin intake over 6 months did not show any statistically significant difference in clinical or the laboratory variables of efficacy when compared to the control group apart from reticulocytic count which was higher in Metformin group throughout the study. Conclusion: Metformin intake, in addition to hydroxyurea, did not yield any extra benefit among patients with NTDT.

## Introduction

Non-transfusion dependent thalassemia (NTDT) refers to a group of thalassemic disorders characterized by infrequent blood transfusions to maintain life, however regular transfusions may be needed under certain circumstances [1]. Fetal hemoglobin (HbF) inducer, Hydroxyurea (HU), has been used as a line of treatment of NTDT. HU enhances the generation of gamma chains that bind with excess alpha globin chains forming HbF, thus minimizing the alpha/beta chain imbalance and ameliorating disease severity [2].

Metformin, a biguanide generally used for treatment of type 2 Diabetes mellitus, was identified as HbF inducer [3]. Metformin increases the expression of the active form of Forkhead box O3 (FOXO3) through increasing its activator, the active form of adenosine monophosphate-activated protein kinase (AMPK). It also reduces the active form of serine/threonine kinase (AKT) thus increasing the levels of FOXO3 in the nucleus where it is active [3]. Stimulation of FOXO3 enhances the synthesis of red blood cells [4–8]. Zhang and colleagues in 2016 observed that hematopoietic stem cells treated with a combination of metformin and HU showed additive HbF induction compared to HU alone [9].

Whereas the effect of metformin on inducing HbF in vitro has been thoroughly studied, to the best of our knowledge its effect in vivo has not yet been explored. The aim of our study was to assess the effectiveness and safety of metformin as an inducer of HbF, in combination with hydroxyurea, among Egyptian patients with NTDT over a period of 6 months.

## Patients and Methods

### Study Design

This is a prospective interventional case–control study that was conducted at the Pediatric Hematology Clinic of Cairo University Children Hospital. The study protocol was approved by Faculty of Medicine, Cairo University and it complies with the 2013 modification of Helsinki declaration [10]. Patients were enrolled in the study after obtaining willingly written informed consent from the adult patients (above 18 years of age) and from the parents or guardians of the adolescent participants (12 to 17 years of age).

### Patients

Inclusion criteria of enrolled subjects were male or female patients aged > 12 years with confirmed diagnosis of NTDT (defined as thalassemia patients who do not require frequent blood transfusions for survival, despite regular transfusions may be needed under certain conditions) [12]. All patients were on a stable fixed dose of HU over the last 3 months preceding the onset of study. We excluded patients who received red blood cell transfusion therapy in the last 3 months, had serum creatinine ˃ 1.4 mg/dl, liver enzymes (ALT and AST) ˃ 3 times upper limits of normal, patients with diabetes mellitus who are receiving other antidiabetic medications and patients with chronic hypoxemic conditions associated with lactic acidosis, such as cardiovascular, renal, hepatic and pulmonary disease. NTDT patients who were receiving fixed dose of HU for at least 3 months as part of standard of care were included as a control group.

One group (Metformin group), fulfilling inclusion and exclusion criteria, started taking Metformin or dimethyl biguanidein (in the form of Cidophage tablets containing 500, 850 and 1000 mg of Metformin HCL produced by CID Co-EGYPT) for a duration of six months as follows:

- Starting dose 500 mg/dose, twice daily for one week.
- The dose was increased to 750 mg/dose taken twice a day (total dose 1500 mg/day) starting from 2nd week and till the end of the 3^rd^ month.
- After 3 months on metformin at 1500 mg/day, the dose was increased by 500 mg per day every week until a maximum dose of 2500 mg/day [11].

All patients on Metformin continued at a stable dose of Hydroxyurea throughout the study duration. The Metformin group was compared to a control group. The drug was dispensed monthly free of charge by the unit's pharmacist.

Participants who received at least one dose of metformin were assessed for safety. All adverse events (related or unrelated to drug) as nausea, GIT disturbances, chest discomfort, weight loss, hypoglycemia, lactic acidosis during study period were thoroughly reviewed and recorded during follow up visits at baseline, 3- and 6-months visits. Serious adverse events were reported within 24 h.

Age and sex matched NTDT patients receiving a stable fixed dose of hydroxyurea throughout the study duration as part of standard care were enrolled as control group.

A thorough clinical examination was performed, medication compliance and adverse events were assessed, transfusion history was checked, and laboratory tests in the form of hemoglobin electrophoresis, complete blood picture, reticulocytic count, liver enzymes, lactate dehydrogenase, bilirubin, kidney function tests, and random blood sugar were done at baseline, 3 and 6 months of the study.

### End Point and Assessments

The primary endpoint was to detect the effectiveness of metformin represented as change in HbF percentage and total hemoglobin levels by end of six months of the study. The secondary endpoint was a change in blood transfusion requirements from baseline. Frequency of all adverse events (related or unrelated to metformin) during the study period were reported.

### Statistical Analysis

Data were collected, coded, revised and entered into the Statistical Package for Social Science (IBM SPSS) version 20. The data were presented as number and percentages for the qualitative data, mean, standard deviations and ranges for the quantitative data with parametric distribution and median with inter quartile range (IQR) for the quantitative data with non-parametric with quantitative data and non-parametric distribution. *Paired t-test* was used in the comparison between two groups with quantitative data for before and after and parametric distribution and *Wilcoxon Rank test* was used in the comparison between two groups with quantitative data foe before and after and non-parametric distribution. The confidence interval was set to 95% and the margin of error accepted was set to 5%. *P*-value was considered significant if < 0.05.

## Results

Patients were screened for eligibility, and 43 were enrolled of whom 21 were assigned to the Metformin group (GroupA) and 22 to the Control group (Group B).

One patient from the Metformin group withdrew consent four months from the start due to weight loss. This patient was excluded from the study and the analysis.

### Baseline Data of Included Subjects

Demographics of both groups were comparable as shown in Table 1. All patients at study onset were on Folic acid, L-carnitine and Vitamin D and Hydroxyurea as standard of care.

**Table 1:** Baseline demographic, laboratory, and treatment data of studied NTDT patients

	Metformin group (n = 20)	Control group (n = 22)	*P*-value
*Demographic data*			
Male (No., %)	10 (50)	9 (40.9)	0.554
Age (years) (Mean ± SD)	15 ± 3.23	13.77 ± 2.45	0.122
*Laboratory data*			
HbF (%) (Median, IQR)	22.55 (15.25–41.55)	22 (14.9–31.5)	0.98
Hemoglobin (g/dl) (Mean ± SD)	7.94 ± 1.28	7.49 ± 0.84	0.262
WBC (× 10^3^/uL) (Mean ± SD)	8.82 ± 5.09	10.81 ± 10.94	0.521
Platelets (× 10^3^/uL) (Mean ± SD)	659.4 ± 308.73	563.64 ± 387.24	0.217
Reticulocytic count (%)(Mean ± SD)	5.45 ± 2.39	3.80 ± 1.50	0.027*
TSB (mg/dL) (Mean ± SD)	1.94 ± 0.56	1.92 ± 0.66	0.928
DSB (mg/dL) (Mean ± SD)	0.4 ± 0.13	0.42 ± 0.21	0.897
LDH(U/L) (Mean ± SD)	389.2 ± 134.23	382.95 ± 144.00	0.650
AST(U/L) (Mean ± SD)	37.10 ± 13.92	33.5 ± 20.77	0.164
ALT(U/L) (Mean ± SD)	22.65 ± 17.4	19.59 ± 8.51	0.930
Creatinine (mg/dL) (Mean ± SD)	0.53 ± 0.14	0.49 ± 0.111	0.356
RBS (mg/dL) (Mean ± SD)	89.80 ± 11.90	90.91 ± 10.81	0.753
*Treatment*			
Hydroxyurea dose(mg/kg/day) (mean ± SD)	17.9 ± 6.34	19.27 ± 3.09	0.463

*HbF* fetal hemoglobin, *WBC* white blood cells, *BUN* blood urea nitrogen, *RBS* random blood sugar, *TSB* total serum bilirubin, *DSB* direct serum bilirubin, *LDH* lactate dehydrogenase, *AST* aspartate transaminase, *ALT* alanine transaminase, *IQR* interquartile range

*P-value considered significant if < 0.05

At baseline, there was no statistically significant difference regarding HbF, hematological and biochemical parameters between both groups apart from reticulocytic count which was significantly higher (*p* = 0.027) among the metformin group as shown in Table 1.

### Comparison of Hematological and Biochemical Parameters of Both Groups

All patients in the metformin group continued treatment till end of study except for one patient who stopped metformin and withdrew consent at 4 months after starting medication due to significant weight loss. This patient was excluded from the study and analysis.

Table 2 shows comparison of laboratory parameters of both groups at 3- and 6-months visits. At three months of treatment, there was no statistically significant difference between both groups regarding HbF, hematological parameters or hemolytic markers apart from reticulocytic count which was significantly higher among the Metformin group (*p* value 0.020), while at the end of the study, reticulocytic count was significantly higher and white blood cells and random blood sugar (RBS) were significantly lower among metformin group (*p* = 0.015, 0.038 and 0.025 respectively). At end of study, though slightly higher among patients in metformin group, HbF level did not reach level of significance (*p* = 0.801).

**Table 2:** Comparison of laboratory parameters of all subjects at 3 and 6 months of the study

	Metformin Group (n = 20)	Control Group (n = 22)	*P*-value
*At 3 months*			
HbF (%) (Median, IQR)	21.8 (16.1–41.35)	21.3 (15.2–39.1)	0.870
WBC (× 10^3^/uL)	10.12 ± 9.22	10.30 ± 9.21	0.279
Hemoglobin(g/dL)	7.83 ± 1.24	7.63 ± 0.60	0.522
Platelets (× 10^3^/uL)	532.95 ± 298.23	443.73 ± 189.43	0.296
Reticulocytic count (%)	6.45 ± 3.01	4.11 ± 1.26	0.020*
TSB (mg/dL)	2.10 ± 0.57	2.05 ± 0.65	0.659
DSB (mg/dL)	0.51 ± 0.15	0.44 ± 0.17	0.202
LDH(U/L)	351.50 ± 106.15	374.82 ± 128.29	0.910
AST(U/L)	34.80 ± 18.53	31.82 ± 11.62	1.000
ALT(U/L)	25.80 ± 19.43	19.23 ± 5.68	0.641
Creatinine(mg/dL)	0.54 ± 0.11	0.53 ± 0.12	0.784
RBS (mg/dL)	93.15 ± 12.92	90.59 ± 8.02	0.441
*At 6 months*			
HbF (%) (Median, IQR)	25.7 (15.53–40.35)	21.4 (14.6–42.58)	0.801
WBC (× 10^3^/uL)	8.00 ± 4.63	10.31 ± 7.52	0.038*
Hemoglobin(g/dL)	7.99 ± 1.17	7.59 ± 0.79	0.384
Platelets (× 10^3^/uL)	538.65 ± 305.58	430.95 ± 180.49	0.320
Reticulocytic count (%)	5.88 ± 2.45	4.05 ± 1.21	0.015*
TSB (mg/dL)	2.21 ± 0.68	1.90 ± 0.54	0.122
DSB (mg/dL)	0.47 ± 0.16	0.46 ± 0.20	0.611
LDH(U/L)	364.10 ± 121.29	377.50 ± 136.03	0.950
AST(U/L)	35.25 ± 15.18	31.86 ± 8.00	0.940
ALT(U/L)	25.80 ± 21.72	21.45 ± 7.33	0.289
Creatinine (mg/dL)	0.52 ± 0.12	0.54 ± 0.11	0.463
RBS (mg/dL)	83.35 ± 12.49	92.68 ± 13.37	0.025*

*HbF* fetal hemoglobin, *WBC* white blood cells, *RBS* random blood sugar, *TSB* total serum bilirubin, *DSB* direct serum bilirubin, *LDH* lactate dehydrogenase, *AST* aspartate transaminase, *ALT* alanine transaminase, *IQR* interquartile range

*P-value considered significant if < 0.05

### Changes in the Metformin Group

Comparing hematological and biochemical parameters among metformin group before and after treatment showed that there was no significant difference regarding hematological, biochemical and hemolytic parameters (Table 3).

**Table 3:** Comparison of hematological and biochemical parameters before and after metformin therapy within metformin group (n = 20)

	Before metformin	After metformin	*P*-value
HbF (%) (Median, IQR)	22.5 (15.13–42.23)	25.7 (15.53–40.35)	0.411
WBC (× 10^3^/uL)	8.82 ± 5.09	8.00 ± 4.63	0.225
Hemoglobin (g/dL)	7.94 ± 1.28	7.99 ± 1.17	0.351
Platelets (× 10^3^/uL)	659.40 ± 308.73	538.65 ± 305.58	0.156
Reticulocytic count (%)	5.45 ± 2.39	5.88 ± 2.45	0.284
TSB (mg/dL)	1.94 ± 0.56	2.21 ± 0.68	0.055
DSB (mg/dL)	0.40 ± 0.13	0.47 ± 0.16	0.082
LDH(U/L)	389.20 ± 134.23	364.10 ± 121.29	0.920
AST(U/L)	37.10 ± 13.92	35.25 ± 15.18	0.370
ALT(U/L)	22.65 ± 17.14	25.80 ± 21.72	0.852
Creatinine (mg/dL)	0.53 ± 0.14	0.52 ± 0.12	0.673
RBS (mg/dL)	89.80 ± 11.90	83.35 ± 12.49	0.133

*HbF* fetal hemoglobin, *WBC* white blood cells, *RBS* random blood sugar, *TSB* total serum bilirubin, *DSB* direct serum bilirubin, *LDH* lactate dehydrogenase, *AST* aspartate transaminase, *ALT* alanine transaminase, *IQR* interquartile range

*P-value considered significant if < 0.05

### Transfusions Among Studied Groups During Study

Five patients were transfused in the metformin group as follows: two patients were transfused once, two patients received blood twice and one patient received blood three times.

One patient in the control group received blood transfusion once. There was no significant difference between both groups (*p* = 0.058).

### Safety Parameters Among the Metformin Group

None of the patients in the metformin group had any serious adverse events during the study. Adverse events included a mild form of diarrhea and vomiting in four patients during the first week of the study on a Metformin dose of 1000 mg/day and no action was taken. Another patient developed mild thrombocytopenia on the second month of the study that was not related to Metformin, which led to temporarily stopping of hydroxyurea for one month and resumed at a lower dose.

## Discussion

Patients with NTDT do not require lifelong blood transfusions to maintain life, however, regular transfusions may be needed under certain circumstances [1]. It has been suggested that metformin, in vitro, can induce HbF in stem cells by activating FOXO3 [3]. However, evidence in vivo is still lacking [12]**.** To the best of our knowledge, this is the only in vivo study that assessed the effectiveness and safety of metformin on induction of HbF in Egyptian patients with NTDT over a period of six months.

By the end of our study, no significant difference in mean HbF level between both groups was noted. This could be due to the presence of other possible genetic modifiers affecting HbF level. Although RBC acts as a proper reservoir for metformin, yet the level in stem cells is not known or assessed [13]. It is possible that the desired metformin concentration is not achieved in vivo [3].

There was no significant rise in HbF within the Metformin group by the end of the study. This is in line with the retrospective in vivo study conducted on three patients with polycystic ovaries and 4 patients with type 2 diabetes mellitus taking metformin for at least 6 months and showed no rise in HbF levels [12]. However, this contradicts the data by one in vitro study where HbF increased by two-folds after two weeks of cell culture with 50 mM of metformin [9].

White blood cell count (WBC) was significantly lower in the Metformin group at the end of the study when compared with the control group. This is in agreement with one study conducted on women with polycystic ovaries and have found out that WBC count have significantly reduced at the end of a six month period of using Metformin [14]. In a study by Ibanz et al., it has been concluded that metformin can reduce the high neutrophilic count among girls with hyperinsulinemic hyperandrogenism [15].

Reticulocyte count was significantly higher in metformin group compared to the control group at end of study. Several case studies report metformin induced hemolytic anemia few days after starting metformin [16–19]. This finding was also evident at baseline before starting metformin treatment and could be attributed to the severity of disease among metformin group.

Random blood sugar was significantly lower in metformin group at end of the study when compared to control group. However, it was not clinically significant and was asymptomatic. Metformin alone is rarely associated with hypoglycemia. Hypoglycemia in patients using metformin may occur in association with vigorous physical activity or fasting [20].

Metformin is generally regarded as a safe drug that has been in the market for more than sixty years. Its common side effects include diarrhea, nausea, vomiting, abdominal discomfort, abnormal stools, myalgia, dyspnea, chest discomfort, chills, flushing and palpitation. This is consistent with our reported adverse events among our study group. The major previously reported adverse event is that metformin causes subclinical increases in lactic acid and appears to cause lactic acidosis in extreme overdose. Metformin use is not advisable in patients with risk factors for lactic acidosis, such as hepatic impairment, heart failure, and chronic kidney disease [21]. None of our patients had any symptoms suggestive of lactic acidosis.

Limitations to our work include small sample size and relatively short duration of follow up. Data on the effect of metformin in NTDT in vivo remains limited. More long-term multicenter studies are recommended.

In conclusion, metformin seemed not to have any additional HbF inducing effect to HU when compared to patients on HU alone.

## Data Availability

The datasets used and analyzed during the current study are available from the corresponding author on reasonable request.
